# Functional fitness tests and their association with upper-limb isokinetic strength in older adults

**DOI:** 10.1007/s40520-026-03406-3

**Published:** 2026-05-08

**Authors:** Carlos Babiloni-Lopez, Javier Gene-Morales, Alvaro Juesas, Pablo Jiménez-Martínez, Pedro Gargallo-Bayo, Agustín Jerez-Martínez, Juan C. Colado

**Affiliations:** 1 Faculty of Humanities and Social Sciences, Department of Education, University Jaume I, Av. Vicent Sos Baynat, Castellon, 12071 Spain; 2 Research Group in Prevention and Health in Exercise and Sports (PHES), Department of Physical Education and Sports, University of Valencia, Valencia, 46010, Spain; 3 Department of Education Sciences, CEU Cardenal Herrera University, Castellón, 46115 Spain; 4 Department of Health Research, ICEN Research Center, Santa Cruz de Tenerife, 38002 Spain

**Keywords:** Aged, Muscle strength, Exercise test, Physical functional performance, Regression analysis, Reference values

## Abstract

**Purpose:**

This study investigated the association between commonly used functional fitness tests (i.e., Up-and-Go Test [UGT], 30-Second Chair Stand Test [30CS], 30-Second Arm Curl Test [30AC], and 6-Minute Walk Test [6MWT]) and upper-limb isokinetc strength (elbow and shoulder flexion-extension isokinetic absolute and relative strength at 60º/s and 180º/s) in older adults. It was hypothesized that upper-limb-specific tests would show stronger associations, that elbow strength would be better explained than shoulder strength, and that higher angular velocities would demonstrate stronger associations.

**Methods:**

Three hundred and twenty-seven apparently healthy older adults (247 women and 80 men; age: 69.27 ± 5.62) enrolled in this cross-sectional study. Isokinetic concentric strength was assessed before functional fitness tests.

**Results:**

Small to moderate correlations were observed between functional fitness tests and shoulder and elbow isokinetic strength. Better performance in the UGT, 30AC, and 6MWT was associated with greater strength (*r* = 0.11–0.46); whereas the 30CS showed inconsistent correlations (*r* = -0.13–0.30). Regression models including functional fitness tests, age, and sex explained a substantial proportion of the variance, with higher values for elbow (*R*^*2*^ = 0.34–0.55) than shoulder strength (*R*^*2*^ = 0.12–0.42). No consistent pattern of stronger associations at higher angular velocities was identified.

**Conclusions:**

Upper-limb isokinetic strength is associated with functional fitness in older adults, although associations were generally small to moderate. Functional fitness, together with age and sex, explained up to 55% of the variance. These findings suggest that functional fitness tests may provide indirect and complementary information related to upper-limb strength in settings where isokinetic dynamometry is not available, but they should not be considered surrogate measures of muscle strength.

**Supplementary Information:**

The online version contains supplementary material available at 10.1007/s40520-026-03406-3.

## Highlights

Functional fitness tests are associated with upper-limb isokinetic strength in older adults.

6MWT showed the strongest associations with elbow and shoulder strength.

Elbow strength showed a higher explained variance than shoulder strength.

**Supplementary Information:**

The online version contains supplementary material available at 10.1007/s40520-026-03406-3.

## Introduction

The global population is aging rapidly, with the number of individuals aged 60 years and older expected to double by 2050 [1]. This demographic shift presents significant challenges for healthcare systems, as age-related declines in physical function are closely associated with loss of independence, reduced quality of life, and increased risk of adverse outcomes, including falls, fractures, and chronic diseases [2]. In this context, accurate and accessible assessment of physical function is essential for clinical decision-making and functional monitoring in older adults.

Muscle strength is a key determinant of functional capacity in older adults and a central feature of conditions such as sarcopenia, which is characterized by progressive loss of muscle mass and function [3, 4]. Current consensus definitions emphasize that low muscle strength is the primary indicator of probable sarcopenia, with handgrip strength commonly used as a practical upper-limb measure for its identification [3, 5]. Although declines in lower-limb strength are strongly associated with impaired mobility and increased fall risk [6], upper-limb strength also plays a critical role in maintaining independence, as it is essential for activities of daily living such as lifting, carrying, pushing, and reaching [7]. Moreover, reduced upper-limb strength has been linked to functional limitations, increased dependency, and poorer health outcomes in older adults [8]. Therefore, assessing upper-limb strength provides complementary, clinically relevant information beyond lower-limb evaluation, particularly for a comprehensive assessment of functional capacity.

Isokinetic dynamometry is widely regarded as the gold standard for assessing muscle strength because it allows precise quantification of force production under controlled conditions and across different angular velocities [9]. In older adults, isokinetic assessment has been used to detect strength deficits and monitor the effects of interventions, demonstrating high reliability and validity [9, 10]. Importantly, the use of different angular velocities (e.g., 60º/s and 180º/s) enables evaluation of distinct neuromuscular characteristics, including maximal strength and velocity-dependent force production, both of which are relevant to functional performance in aging populations [11, 12]. However, despite its methodological advantages, the use of isokinetic dynamometry is limited in most clinical and community settings due to its high cost, time requirements, and need for specialized equipment and trained personnel [13]. These limitations highlight the need for alternative, accessible methods to assess muscle strength in real-world contexts.

Functional fitness tests, such as the Up-and-Go Test (UGT), 30-Second Chair Stand Test (30CS), 30-Second Arm Curl Test (30AC), and 6-Minute Walk Test (6MWT), are widely used in clinical and community environments due to their feasibility, low cost, and strong reliability [14, 15]. These tests assess multiple domains of physical function, including mobility, muscular endurance, and aerobic capacity, and are commonly used to evaluate functional status in older adults. Although they are often assumed to reflect underlying muscular strength, they primarily capture integrated functional performance involving multiple physiological systems, and their specific association with isolated muscle strength remains unclear.

Despite their widespread use, the relationship between functional fitness tests and upper-limb isokinetic strength has not been clearly established. While previous studies have used isokinetic dynamometry to assess upper-limb function in specific contexts [16, 17], evidence linking these measures with widely used functional fitness tests remains scarce. Previous research has predominantly focused on lower-limb strength and its relationship to mobility and fall risk, whereas evidence on upper-limb strength remains limited. However, no previous study has systematically examined whether commonly used functional fitness tests can serve as valid proxies for upper-limb isokinetic strength in older adults. Addressing this gap is clinically relevant, as identifying simple, accessible tools for assessing upper-limb strength may improve screening and monitoring of functional decline in settings where isokinetic dynamometry is unavailable.

Therefore, the present study aimed to investigate the association between commonly used functional fitness tests (UGT, 30CS, 30AC, and 6MWT) and upper-limb isokinetic strength (absolute and relative elbow and shoulder flexion-extension) at two angular velocities (60º/s and 180º/s) in older adults. We hypothesized that: (1) upper-limb-specific tests (30AC) would show stronger associations with upper-limb isokinetic strength than lower-limb-oriented tests (UGT, 30CS, 6MWT); (2) elbow isokinetic strength would be more strongly associated than shoulder strength due to the greater movement specificity and segmental involvement in the 30AC; and (3) associations would be stronger at 180º/s compared to 60º/s, due to their closer relationship with functional performance.

## Materials and methods

### Experimental design and ethical approval

This cross-sectional study (clinical trial registration: NCT06618469) examined the association between functional fitness tests (UGT, 30CS, 30AC, and 6MWT) and absolute and relative upper-limb isokinetic strength (elbow and shoulder flexion-extension at 60º/s and 180º/s) in older adults. In addition, cross-sectional regression models were used to describe the association between functional fitness test performance and upper-limb isokinetic strength. The study was reported in accordance with the STROBE (Strengthening the Reporting of Observational Studies in Epidemiology, see Supplementary Material Table 1) guidelines for cross-sectional studies [18]. All participants provided written informed consent. The protocol was approved by the Ethics Committee of the University of ***blinded for peer review*** (approval: ***blinded for peer review***), and conducted in accordance with the Declaration of Helsinki.

Participants attended the laboratory once. To minimize the effects of neuromuscular and cardiorespiratory fatigue [19], anthropometric measurements were obtained first, followed by isokinetic strength assessment and functional fitness tests. Within the functional battery, the agility and dynamic balance (i.e., UGT) were assessed before strength (30CS and 30AC) and aerobic endurance (6MWT). Sessions lasted 60–80 min and were conducted in the morning under controlled environmental conditions (22–24 °C, 55% humidity). The study design followed previously reported procedures [20].

### Participants

An a priori sample size analysis (G*Power 3.1) [21] for multiple linear regression (fixed model, R^2^ deviation from zero, six independent variables) indicated that 239 participants were required to achieve 95% power (α = 0.05; f^2^ = 0.09). The selected effect size was based on a previous cross-sectional regression analysis examining lower-limb strength [20]. Many additional participants responded to the call and attended the laboratory. A larger sample was ultimately included to improve statistical precision and account for potential population heterogeneity.

A total of 327 healthy older adults (247 women and 80 men) participated. Inclusion criteria were: (1) age ≥ 60 years; (2) no structured exercise participation in the previous six months; (3) independent ambulation; and (4) absence of severe diseases. Exclusion criteria included: (1) cardiovascular, neurological, respiratory, or musculoskeletal conditions affecting test performance; (2) body weight changes > 10% in the previous year; and (3) medications or supplements affecting neuromuscular performance. Participants were instructed to maintain their normal daily routine, avoid food intake for two hours before testing, and refrain from exercise for 24 h prior to assessment. Recruitment was conducted through advertisements in municipal activity centres for older adults in ***blinded for peer review***.

### Anthropometric measurements

Body mass and body composition were measured using a digital scale (Tanita^®^ BC-418 MA, Tokyo, Japan), and height was measured with a portable stadiometer (Seca T214, Hamburg, Germany).

### Isokinetic strength assessment

Isokinetic strength was assessed using a calibrated Biodex^®^ Multi-Joint System V.4× dynamometer (Biodex Medical System 5 TM, Shirley, NY, USA) with Advantage software (version 3.2). Peak concentric torque (N·m) was recorded during seated shoulder and elbow flexion-extension at angular velocities of 180º/s and 60º/s. The range of motion was set from 10º to 85º for shoulder flexion, and from 15 to 75° for elbow flexion. Relative isokinetic strength was calculated by normalizing peak torque to body mass (N·m.kg^-1^). All assessments were performed on the dominant limb by the same certified examiner to ensure consistency.

Participants completed a standardized 5-minute warm-up consisting of moderate-intensity walking, active stretching, and upper-limb mobility exercises. A familiarization set of continuous concentric contractions lasting 30 s was then performed, followed by one minute of rest. Shoulder and elbow tests were performed in randomized order, with two minutes of rest between exercises. Each movement was evaluated first at 180º/s and then at 60º/s, with one minute of rest between velocities. These angular velocities were selected to assess velocity-dependent force production (180º/s) and maximal strength (60º/s) [12]. For each condition, participants performed five maximal voluntary contractions, and the highest peak torque value was used for analysis. Strong verbal encouragement was provided throughout testing to promote maximal effort.

### Physical fitness assessment

Four tests from the Senior Fitness Test battery [14] were selected based on study objectives, feasibility, and facility availability. All the tests were administered in the same order and performed according to the standardized procedures described in the Senior Fitness Test manual [15]. Two experienced researchers provided instructions and supervision to ensure adherence to the protocol and measurement accuracy. The following tests were performed: (1) UGT, which assesses agility and dynamic balance; (2) 30AC, which reflects upper-limb functional performance and muscular endurance; (3) 30CS, which reflects lower-limb functional performance; and (4) 6MWT, which assesses aerobic endurance. These tests have demonstrated excellent reliability in older adults (intraclass correlation coefficient [ICC] = 0.965–0.988; coefficient of variation [CV] = 1.69–2.80%).

### Statistical analysis

Statistical analyses were performed using SPSS software (version 26.0; IBM Corp., Armonk, NY, USA). Results are presented as mean ± SD, and statistical significance was set at *p* < 0.05. Participants with missing isokinetic strength data were excluded prior to analysis, resulting in a final sample of 327 individuals. Within this sample, missing data in the remaining variables were minimal (≤ 1.8%) and were handled using complete-case analysis, as the low proportion of missingness was considered unlikely to materially affect the results. Extreme values were identified using standardized z-scores (|z| > 3.29), following established recommendations [22]. A small number of extreme observations were detected across variables (with a maximum of four cases in the 30CS). These values were winsorized to the nearest non-outlying value to reduce their influence while preserving sample size. Sensitivity analyses comparing original and winsorized datasets were conducted as a preliminary step to assess the robustness of the results, without materially affecting the overall conclusions.

Independent sample t-tests were used to examine sex differences in descriptive variables. Sex was subsequently included as a predictor variable due to its well-established influence on muscle strength, with consistent evidence showing greater absolute strength in men, particularly in upper-body musculature [23]. Zero-order and partial correlations were conducted to examine associations between functional fitness tests and absolute and relative isokinetic strength. Partial correlations were adjusted for age and sex, given their established influence on muscle strength and their inclusion in the regression models. Correlation coefficients were interpreted as trivial (0.00-0.09), small (0.10–0.29), moderate (0.30–0.49), large (0.50–0.69), very large (0.70–0.89), nearly perfect (0.90–0.99), and perfect (1.0) [24].

Stepwise multiple linear regression analyses were performed to examine the association between functional fitness tests and absolute and relative upper-limb isokinetic strength, and to identify the most parsimonious models. Independent variables included functional fitness test performance, age, and sex. Due to the cross-sectional design, results from these models should be interpreted as associations rather than causal or predictive relationships. To further explore the influence of sex, additional exploratory analyses were conducted separately for women and men using standard linear regression models (enter method), which supported the interpretation of the main findings. The assumptions of linear regression were assessed for all models. Normality of residuals was evaluated using normal P–P plots, while homoscedasticity and linearity were examined through visual inspection of scatterplots of standardized residuals versus predicted values. Independence of residuals was evaluated using the Durbin-Watson statistic, and multicollinearity was assessed using variance inflation factors (VIF), with values considered acceptable when VIF < 5 and Durbin–Watson values were close to 2 [25].

Measurement reliability was evaluated in a subsample of 33 participants assessed on three non-consecutive days (i.e., *k* = 3). ICC and 95% confidence intervals (CI) were calculated using a two-way mixed-effects model with absolute agreement [26]. Coefficients of variation (CV) were calculated to assess measurement consistency.

## Results

### Baseline characteristics

**Fig. 1:**
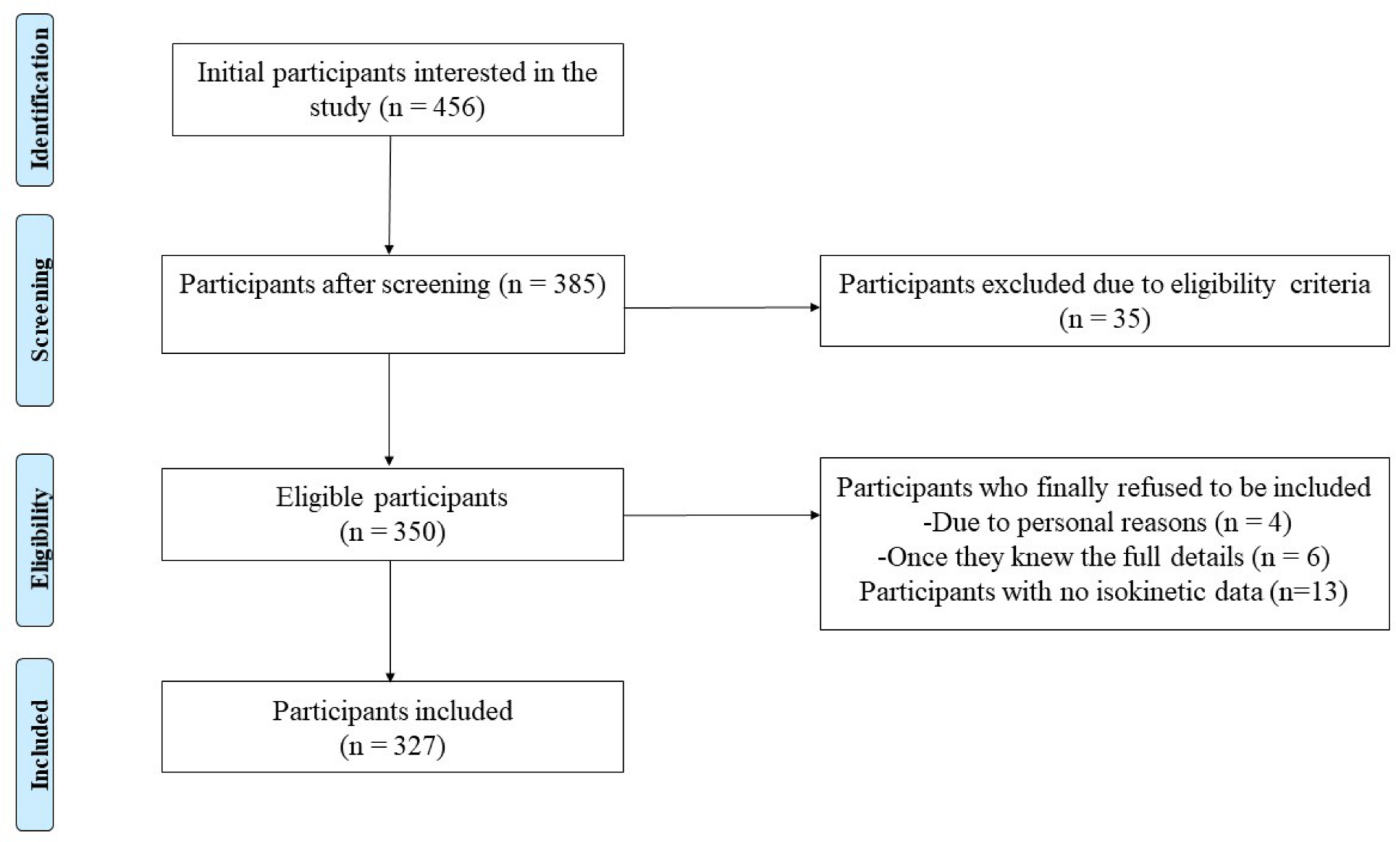
Flow chart illustrating participants’ enrollment

An initial sample of 456 participants was recruited, resulting in 327 participants (247 females and 80 males) after exclusions and withdrawals (Fig. 1).

Table 1 presents age, anthropometric characteristics, physical fitness test performance, and upper-limb isokinetic strength (absolute and relative) for the total sample, stratified by sex. Differences between men and women were observed across most variables. Normative percentile values (10th, 25th, 50th, 75th, and 90th ) for upper-limb isokinetic strength stratified by age and sex were also calculated and are provided as supplementary material (Tables S2-S3).

**Table 1:** Main characteristics of the older female and male study participants

Variables	*Both Sexes (n = 327)*	*Women (n = 247)*	*Men (n = 80)*
** Age (years)**	69.27 ± 5.62	68.93 ± 5.97	70.30 ± 4.28
** Height (m)**	1.57 ± 0.08	1.54 ± 0.06	1.66 ± 0.06*
** Weight (kg)**	70.42 ± 11.17	67.65 ± 10.54	78.46 ± 8.97*
** Body Fat (%)**	40.32 ± 6.78	43.18 ± 4.45	30.99 ± 4.22*
** BMI (kg×m**^**2**^**)**	28.48 ± 3.92	28.42 ± 4.22	28.59 ± 2.82
** Up-and-Go Test (s)**	6.38 ± 1.14	6.46 ± 1.13	6.12 ± 1.14*
**Chair Stand Test (reps)**	13.14 ± 3.71	12.89 ± 3.54	13.89 ± 4.11
** Arm Curl Test (reps)**	17.65 ± 4.68	17.23 ± 4.40	18.97 ± 5.22*
** 6-minute Walk Test (m)**	518.71 ± 72.16	510.06 ± 68.48	546.64 ± 75.54*
**Shoulder Flexion at 180º/s (N·m)**	44.69 ± 19.30	38.92 ± 14.19	65.44 ± 19.35*
**Shoulder Flexion at 60º/s (N·m)**	39.08 ± 17.24	33.94 ± 12.41	57.17 ± 18.35*
**Shoulder Extension at 180º/s (N·m)**	42.22 ± 12.20	38.86 ± 10.12	53.23 ± 11.49*
**Shoulder Extension at 60º/s (N·m)**	40.84 ± 14.70	36.70 ± 12.40	54.30 ± 13.42*
** Elbow Flexion at 180º/s (N·m)**	17.99 ± 7.25	15.32 ± 4.81	26.97 ± 6.35*
** Elbow Flexion at 60º/s (N·m)**	19.89 ± 8.45	16.76 ± 5.30	30.63 ± 7.69*
**Elbow Extension at 180º/s (N·m)**	34.02 ± 11.44	30.19 ± 7.96	47.60 ± 10.62*
** Elbow Extension at 60º/s (N·m)**	38.98 ± 13.78	34.27 ± 8.75	56.46 ± 12.77*
**Relative Shoulder Flexion at 180º/s (N·m/kg** ^**− 1**^ **)**	0.64 ± 0.24	0.58 ± 0.22	0.82 ± 0.23*
**Relative Shoulder Flexion at 60º/s (N·m/kg** ^**− 1**^ **)**	0.56 ± 0.22	0.51 ± 0.19	0.72 ± 0.21*
**Relative Shoulder Extension at 180º/s (N·m/kg** ^**− 1**^ **)**	0.60 ± 0.17	0.58 ± 0.17	0.68 ± 0.17*
**Relative Shoulder Extension at 60º/s (N·m/kg** ^**− 1**^ **)**	0.59 ± 0.20	0.55 ± 0.19	0.70 ± 0.17*
** Relative Elbow Flexion at 180º/s (N·m/kg** ^**− 1**^ **)**	0.26 ± 0.09	0.23 ± 0.07	0.35 ± 0.08*
** Relative Elbow Flexion at 60º/s (N·m/kg** ^**− 1**^ **)**	0.29 ± 0.10	0.25 ± 0.08	0.39 ± 0.09*
** Relative Elbow Extension at 180º/s (N·m/kg** ^**− 1**^ **)**	0.49 ± 0.14	0.45 ± 0.12	0.60 ± 0.13*
** Relative Elbow Extension at 60º/s (N·m/kg** ^**− 1**^ **)**	0.56 ± 0.17	0.51 ± 0.14	0.71 ± 0.15*

*Significant differences between sex (*p* < 0.05). m: metres; kg: kilograms; s: seconds; reps: repetitions: N·m: Newton per metre.

### Relationship between physical fitness tests and upper limb isokinetic strength

The relationships between functional fitness tests and absolute and relative upper-limb isokinetic strength are shown in Table 2. After adjusting for covariates, the magnitudes of the associations were generally reduced. The UGT showed small-to-moderate associations with elbow extension at both velocities. The 30CS demonstrated low and inconsistent associations with shoulder and elbow strength. The 30AC showed stronger associations with elbow movements than with shoulder movements. The 6MWT presented the largest correlations overall, with similar magnitudes for elbow and shoulder strength at both velocities. Relative strength values generally showed stronger associations with the UGT, 30CS, and 6MWT compared with absolute strength, whereas this pattern was not observed for the 30AC. In addition, sex-stratified correlation analyses were conducted as an additional exploratory approach and are presented in the Supplementary Material (Tables S4-S5).

**Table 2:** Associations between functional fitness tests and upper-limb isokinetic strength

Isokinetic upper-limb tests	Correlation coefficient (*r*)			
	Up-and-Go Test	30-second Chair Stand	30-second Arm Curl	6-min Walk Test
Shoulder flexion 180º/s	-0.19** (N.S.)	0.13* (N.S.)	0.26** (0.16**)	0.40** (0.26**)
Shoulder flexion 60º/s	-0.15** (N.S.)	0.15** (N.S.)	0.27** (0.16**)	0.40** (0.25**)
Shoulder extension 180º/s	-0.15** (N.S)	N.S. (N.S.)	0.16** (N.S.)	0.27** (0.12*)
Shoulder extension 60º/s	-0.13** (N.S.)	N.S. (-0.11*)	0.15** (N.S.)	0.21** (N.S.)
Elbow flexion 180º/s	-0.18** (N.S.)	N.S. (N.S.)	0.29** (0.19**)	0.26** (N.S.)
Elbow flexion 60º/s	-0.14** (N.S.)	N.S. (N.S.)	0.23** (0.11*)	0.25** (N.S.)
Elbow extension 180º/s	-0.20** (N.S.)	0.21** (0.16**)	0.28** (0.19**)	0.36** (0.21**)
Elbow extension 60º/s	-0.23** (-0.14*)	0.17** (N.S.)	0.28** (0.18**)	0.40** (0.28**)
Relative shoulder flexion 180º/s	-0.25** (-0.15*)	0.20** (0.13*)	0.24** (0.14*)	0.45** (0.32**)
Relative shoulder flexion 60º/s	-0.19** (N.S.)	0.20** (0.14*)	0.24** (0.13*)	0.44** (0.31**)
Relative shoulder extension 180º/s	-0.18** (-0.11*)	N.S. (N.S.)	N.S. (N.S.)	0.28** (0.18**)
Relative shoulder extension 60º/s	-0.16** (-0.12*)	N.S. (N.S.)	N.S. (N.S.)	0.21** (0.14**)
Relative elbow flexion 180º/s	-0.24** (-0.12*)	0.12* (N.S.)	0.26** (0.14*)	0.29** (N.S.)
Relative elbow flexion 60º/s	-0.18** (N.S.)	N.S. (N.S.)	0.19** (N.S.)	0.28** (N.S.)
Relative elbow extension 180º/s	-0.27** (-0.18**)	0.30** (0.25**)	0.25** (0.16**)	0.41** (0.28**)
Relative elbow extension 60º/s	-0.31** (-0.23**)	0.26** (0.20**)	0.26** (0.16**)	0.46** (0.36**)

Data in parentheses show partial correlations adjusted for age and sex. N.S.: Non-significant. *: *p* < 0.05; **: *p* < 0.01.

### Cross-sectional regression models describing the association between physical fitness tests and upper-limb isokinetic strength

Table 3 presents stepwise regression models for absolute and relative elbow and shoulder flexion-extension at 60º/s and 180º/s. The highest coefficients of determination were observed for absolute elbow flexion at 60º/s and 180º/s (R^2^ = 0.54 and 0.55, respectively) and for elbow extension at 60º/s and 180º/s (R^2^ = 0.56 and 0.48, respectively). These models explained a moderate proportion of the variance in upper-limb isokinetic strength. Based on standardized coefficients (β), sex showed the largest contribution across most models. The 6MWT was also frequently included among the variables retained in the models. Models for relative strength showed R² values ranging from 0.12 (shoulder extension at both velocities) to 0.43 (elbow extension at 60º/s), indicating lower but still meaningful explained variance compared with absolute strength models. The 30AC was included in all absolute strength, regardless of joint and velocity. For relative strength, the 30AC was retained in models for shoulder flexion at both velocities and elbow flexion at 180º/s. A substantial proportion of the explained variance was attributable to sex differences, as reflected by the magnitude of the standardized coefficients. Full regression models used for these analyses are provided in the Supplementary Material (Table S6). Collinearity diagnostics (variance inflation factor, VIF) and model assumptions, including Durbin–Watson statistics, are reported in Table 3.

**Table 3:** Stepwise linear regression models examining the association between functional fitness tests and upper-limb isokinetic strength (absolute and relative values) in older adults

Outcome	*R* ^2^	Independent variables	Standardized β coefficients	DW	Maximum VIF
*Absolute Values*					
Shoulder flexion 180º/s	0.42	Sex, 6MWT, 30AC	0.50, 0.25, 0.11	1.91	1.12
Shoulder flexion 60º/s	0.42	Sex, 6MWT, 30AC	0.50, 0.26, 0.12	1.80	1.13
Shoulder extension 180º/s	0.29	Sex, 6MWT	0.48, 0.16	1.56	1.06
Shoulder extension 60º/s	0.26	Sex	0.51	1.43	1.00
Elbow flexion 180º/s	0.55	Sex, 30AC, 30CS, Age	0.69, 0.25, -0.18, -0.17	1.27	1.67
Elbow flexion 60º/s	0.54	Sex, 30AC, 30CS, Age	0.70, 0.17, -0.14, -0.12	0.49	1.67
Elbow extension 180º/s	0.48	Sex, 6MWT, 30AC	0.58, 0.19, 0.13	1.92	1.13
Elbow extension 60º/s	0.56	Sex, 6MWT, 30AC	0.62, 0.23, 0.11	1.81	1.13
*Relative Values*					
Shoulder flexion 180º/s	0.31	6MWT, Sex, 30AC	0.34, 0.33, 0.10	1.81	1.13
Shoulder flexion 60º/s	0.32	Sex, 6MWT, Age	0.36, 0.31, -0.11	1.78	1.32
Shoulder extension 180º/s	0.12	6MWT, Sex	0.23, 0.21	1.53	1.06
Shoulder extension 60º/s	0.12	Sex,6MWT	0.29, 0.14	1.38	1.06
Elbow flexion 180º/s	0.38	Sex, Age, 30AC	0.56, -0.21, 0.12	1.03	1.10
Elbow flexion 60º/s	0.35	Sex, Age	0.59, -0.17	0.16	1.01
Elbow extension 180º/s	0.34	Sex, 6MWT, 30CS	0.38, 0.27, 0.19	1.78	1.11
Elbow extension 60º/s	0.43	Sex, 6MWT, 30CS	0.45, 0.33, 0.13	1.61	1.11

All regression models were statistically significant (*p* < 0.01). Values of R² represent the proportion of explained variance. β values correspond to standardized regression coefficients. Only variables retained in the final stepwise models are presented.

UGT: Up-and-Go Test; 30CS: 30-Second Chair Stand Test; 30AC: 30-Second Arm Curl Test; 6MWT: 6-Minute Walk Test; DW: Durbin–Watson statistic; VIF: Variance Inflation Factor.

## Discussion

This study examined the associations between functional fitness tests (UGT, 30CS, 30AC, and 6MWT) and upper-limb isokinetic strength (shoulder and elbow flexion-extension at 60º/s and 180º/s) in older adults. To our knowledge, this is the first study to systematically investigate these associations in the upper-limb joints, addressing a gap in a field traditionally focused on lower-body function.

Regarding the first hypothesis, the results were only partially supported. The 6MWT showed the strongest associations across tests (*r* = 0.21 to 0.46), suggesting that global functional capacity has a modest but consistent relationship with upper-limb strength, but with limited ability to accurately estimate individual strength levels. In contrast, the 30AC demonstrated smaller associations with elbow strength (*r* = 0.15 to 0.28), reflecting a more localized contribution related to upper-limb muscular endurance. The UGT showed inverse associations (*r* = -0.31 to -0.13), indicating that greater strength is associated with better overall functional mobility [27]. Conversely, the 30CS showed low and inconsistent associations, suggesting limited relevance for assessing upper-limb strength.

Overall, the magnitude of the associations was small to moderate, indicating that functional fitness tests explain only a limited proportion of the variability in upper-limb isokinetic strength. This is consistent with the regression models (R² = 0.31–0.55), which, although moderate, indicate that a substantial proportion of the variance remains unexplained, limiting the utility of these models for individual-level estimation. These findings highlight the multifactorial nature of muscle strength and suggest that functional fitness tests should not be considered surrogate measures of upper-limb strength, but rather indicators of overall functional performance influenced by multiple physiological systems. Furthermore, given the cross-sectional design, these associations should not be interpreted as causal or predictive relationships.

From a physiological perspective, age-related declines in muscle strength reflect a systemic process affecting both upper and lower limbs [3]. Although lower-limb strength is more directly linked to mobility and fall risk [6], upper-limb strength may reflect overall neuromuscular deterioration and reduced functional reserve [28], which may explain its association with global functional measures.

The relatively stronger associations observed with the 6MWT likely reflect its integrative nature, as it captures cardiovascular, respiratory, and neuromuscular function [29]. In contrast, the 30AC reflects more localized muscular endurance, which may explain its weaker association with peak isokinetic strength. Together, these findings suggest that functional fitness tests capture integrated physiological capacity rather than isolated muscular strength [30].

Regarding the second hypothesis, elbow strength showed stronger associations than shoulder strength. This may be explained by biomechanical differences, as the shoulder joint has greater degrees of freedom and requires more complex neuromuscular control [31], increasing variability in strength measurements. In contrast, the elbow is more mechanically constrained, allowing for more consistent force production [32], particularly in older adults, where variability may be further influenced by joint stiffness and neuromuscular changes [33].

Regression analysis showed that functional fitness tests, together with age and sex, explained a moderate proportion of the variance in upper-limb isokinetic strength. Sex showed the largest contribution across most models (*β* = 0.47–0.70), consistent with well-established differences in muscle strength between men and women [34, 35]. Importantly, a substantial proportion of the explained variance appears attributable to sex differences, suggesting that the models partly reflect underlying biological differences rather than purely functional relationships. In this context, sex should be considered a key determinant of upper-limb strength. Age also contributed, reflecting the well-established decline in muscle function with aging [20]. The 30AC showed a relatively greater contribution in models involving elbow strength, supporting the role of movement specificity.

The hypothesis of stronger associations at higher velocities (180º/s) was only partially supported. Although slightly stronger associations were observed in some cases (particularly 6MWT), meaningful relationships were also present at 60º/s. Age-related atrophy of fast-twitch fibers may increase variability in rapid contractions, potentially explaining the lack of consistent differences between velocities [36, 37].

From a clinical perspective, isokinetic dynamometry remains a valuable tool, as it provides a precise assessment of muscle strength across different velocities, enabling detailed characterization of neuromuscular function. Monitoring isokinetic strength may therefore be useful for identifying strength deficits, evaluating interventions, and understanding functional decline in older adults.

From a practical perspective, functional fitness tests may provide complementary information on upper-limb strength. Although they cannot replace isokinetic assessment, they may be useful for screening or monitoring in settings where laboratory measurements are unavailable. A multidimensional test battery combining upper- and lower-limb assessments may therefore provide a more comprehensive evaluation of functional capacity. Additionally, relative strength showed stronger associations with functional performance than absolute strength, highlighting the importance of normalizing strength to body mass in populations with high anthropometric variability.

This study has several strengths, including the use of a gold-standard isokinetic dynamometer, a large sample size, and multiple functional fitness tests. However, limitations should be acknowledged. The cross-sectional design precludes causal inference, and the predominance of women may limit generalizability. A considerable proportion of the explained variance was attributable to sex differences, which may limit interpretation as purely functional relationships. Furthermore, although stepwise regression was used to identify parsimonious models, this approach has inherent limitations, including potential overfitting and instability in variable selection. Nevertheless, the consistency of the findings across models supports their robustness. Future research should include more diverse samples and longitudinal designs to clarify these relationships. Additionally, normative percentile values are provided in the supplementary material and may serve as reference data for future studies, although they should be interpreted with caution due to the smaller subgroup sample sizes.

## Conclusion

The present study shows that functional fitness test performance (UGT, 30CS, 30AC, and 6MWT) is associated with shoulder and elbow strength at both 60º/s and 180º/s. Although the observed associations were generally small to moderate, functional fitness tests, together with age and sex, explained a meaningful proportion of the variance in upper-limb strength, particularly for elbow movements. However, a substantial share of this variance is attributable to sex differences. Therefore, functional fitness tests should be considered complementary tools that reflect integrated physical performance rather than direct indicators of isolated muscle strength.

## Electronic Supplementary Material

Below is the link to the electronic supplementary material.

Supplementary Material 1

Supplementary Material 2

Supplementary Material 3

Supplementary Material 4

Supplementary Material 5

Supplementary Material 6

## Data Availability

The datasets generated during and analyzed during the current study are available from the corresponding author upon reasonable request.

## Abbreviations

UGT: Up-and-Go Test
30CS: 30-Second Chair Stand Test
30AC: 30-Second Arm Curl Test
6MWT: 6-Minute Walk Test
ICD-10-CM: International Classification of Diseases, 10th Revision, Clinical modification
STROBE: Strengthening the reporting of observational studies in epidemiology
SD: Standard deviation
ICC: Intraclass correlation coefficient
CI: Confidence interval
CV: Coefficient of variation
DW: Durbin watson test
m: Meter
kg: Kilogram
s: Seconds
reps: Repetitions
N·m: Newton per meter
