# Supplementary material for: Functional fitness tests and their association with upper-limb isokinetic strength in older adults
Source: Aging Clin Exp Res. 2026 May 8;38(1):162. doi: 10.1007/s40520-026-03406-3 (PMC13357388; doi:10.1007/s40520-026-03406-3)
Supplement: Supplementary file 1 — Supplementary Material 1 [file 40520_2026_3406_MOESM1_ESM.docx]

**Table S1.** STROBE Statement—Checklist of items that should be included in reports of *cross-sectional studies*

|  | Item No | Recommendation |
| --- | --- | --- |
| **Title and abstract** | 1 | (*a*) Indicate the study’s design with a commonly used term in the title or the abstract **/LINE 1** |
|  |  | (*b*) Provide in the abstract an informative and balanced summary of what was done and what was found **/LINE 30-52** |
| Introduction | | |
| Background/rationale | 2 | Explain the scientific background and rationale for the investigation being reported **/LINE 81-134** |
| Objectives | 3 | State specific objectives, including any prespecified hypotheses **/LINE 136-144** |
| Methods | | |
| Study design | 4 | Present key elements of study design early in the paper **/LINE 148-158** |
| Setting | 5 | Describe the setting, locations, and relevant dates, including periods of recruitment, exposure, follow-up, and data collection **/LINE 159-165** |
| Participants | 6 | (*a*) Give the eligibility criteria, and the sources and methods of selection of participants **/LINE 173-181** |
| Variables | 7 | Clearly define all outcomes, exposures, predictors, potential confounders, and effect modifiers. Give diagnostic criteria, if applicable **/LINE 148-152** |
| Data sources/ measurement | 8* | For each variable of interest, give sources of data and details of methods of assessment (measurement). Describe comparability of assessment methods if there is more than one group **/LINE 182-214** |
| Bias | 9 | Describe any efforts to address potential sources of bias **/LINE 216-250.** |
| Study size | 10 | Explain how the study size was arrived at **/LINE 167-172** |
| Quantitative variables | 11 | Explain how quantitative variables were handled in the analyses. If applicable, describe which groupings were chosen and why **/LINE 216-250** |
| Statistical methods | 12 | (*a*) Describe all statistical methods, including those used to control for confounding **/LINE 216-250** |
|  |  | (*b*) Describe any methods used to examine subgroups and interactions **/LINE 240-242** |
|  |  | (*c*) Explain how missing data were addressed **/LINE 219-226** |
|  |  | (*d*) If applicable, describe analytical methods taking account of sampling strategy **/NOT NECESSARY** |
|  |  | (*e*) Describe any sensitivity analyses **/Line 240-242** |
| Results | | |
| Participants | 13* | (a) Report numbers of individuals at each stage of study—eg numbers potentially eligible, examined for eligibility, confirmed eligible, included in the study, completing follow-up, and analysed **/LINE 253-259; Figure 1; Table 1** |
|  |  | (b) Give reasons for non-participation at each stage **/Figure 1** |
|  |  | (c) Consider use of a flow diagram **/Figure 1** |
| Descriptive data | 14* | (a) Give characteristics of study participants (eg demographic, clinical, social) and information on exposures and potential confounders **/Table 1 and Tables S2-S3** |
|  |  | (b) Indicate number of participants with missing data for each variable of interest **/Line 223-226** |
| Outcome data | 15* | Report numbers of outcome events or summary measures **/LINE 263-292; Tables 2-3** |
| Main results | 16 | (*a*) Give unadjusted estimates and, if applicable, confounder-adjusted estimates and their precision (eg, 95% confidence interval). Make clear which confounders were adjusted for and why they were included **/Tables 2-3** |
|  |  | (*b*) Report category boundaries when continuous variables were categorized **/NOT NECESSARY** |
|  |  | (*c*) If relevant, consider translating estimates of relative risk into absolute risk for a meaningful time period **/NOT NECESSARY** |
| Other analyses | 17 | Report other analyses done—eg analyses of subgroups and interactions, and sensitivity analyses **/Tables S2-S6** |
| Discussion | | |
| Key results | 18 | Summarise key results with reference to study objectives **/LINE 295-299** |
| Limitations | 19 | Discuss limitations of the study, taking into account sources of potential bias or imprecision. Discuss both direction and magnitude of any potential bias **/LINE 382-394** |
| Interpretation | 20 | Give a cautious overall interpretation of results considering objectives, limitations, multiplicity of analyses, results from similar studies, and other relevant evidence **/LINE 301-363** |
| Generalisability | 21 | Discuss the generalisability (external validity) of the study results **/LINE 365-380** |
| Other information | | |
| Funding | 22 | Give the source of funding and the role of the funders for the present study and, if applicable, for the original study on which the present article is based **/Title Page** |

*Give information separately for exposed and unexposed groups.

**Note:** An Explanation and Elaboration article discusses each checklist item and gives methodological background and published examples of transparent reporting. The STROBE checklist is best used in conjunction with this article (freely available on the Web sites of PLoS Medicine at http://www.plosmedicine.org/, Annals of Internal Medicine at http://www.annals.org/, and Epidemiology at http://www.epidem.com/). Information on the STROBE Initiative is available at www.strobe-statement.org.
