# Supplementary material for: Functional fitness tests and their association with upper-limb isokinetic strength in older adults
Source: Aging Clin Exp Res. 2026 May 8;38(1):162. doi: 10.1007/s40520-026-03406-3 (PMC13357388; doi:10.1007/s40520-026-03406-3)
Supplement: Supplementary file 2 — Supplementary Material 2 [file 40520_2026_3406_MOESM2_ESM.docx]

| **Table S2.** Normative percentile values for isokinetic shoulder strength (absolute and relative). | | | | | | | | | | | | |
| --- | --- | --- | --- | --- | --- | --- | --- | --- | --- | --- | --- | --- |
|  |  | *Female* | | | | |  | *Male* | | | | |
| **Percentiles** | ***n*** | **10th** | **25th** | **50th** | **75th** | **90th** | ***n*** | **10th** | **25th** | **50th** | **75th** | **90th** |
| **Age** |  | *Isokinetic Shoulder Flexion* *at 180º/s [N·m] (Relative Isokinetic Shoulder Flexion at 180º/s [N·m/kg])* | | | | | | | | | | |
| **60-64** | 59 (18.04%) | 21.8 (0.37) | 33.7 (0.53) | 46.0 (0.69) | 56.0 (0.80) | 60.1 (0.93) | 5 (1.52%) | 32.2 (0.41) | 41.6 (0.53) | 57.7 (0.79) | 66.5 (0.90) | - |
| **65-69** | 79 (24.15%) | 20.4 (0.30) | 28.3 (0.43) | 40.5 (0.59) | 52.5 (0.76) | 61.8 (0.92) | 32 (9.78%) | 39.5 (0.48) | 57.1 (0.64) | 73.7 (0.87) | 82.6 (1.06) | 102.0 (1.24) |
| **70-74** | 61 (18.65%) | 17.8 (0.27) | 25.7 (0.35) | 33.7 (0.51) | 45.5 (0.75) | 55.0 (0.83) | 29 (8.86%) | 26.5 (0.39) | 50.3 (0.61) | 59.0 (0.78) | 78.5 (0.97) | 87.8 (1.18) |
| **75-79** | 34 (10.39%) | 18.9 (0.26) | 27.2 (0.38) | 34.6 (0.46) | 44.3 (0.59) | 64.4 (0.94) | 13 (3.97%) | 36.3 (0.53) | 52.2 (0.72) | 70.5 (0.87) | 76.2 (0.96) | 80.8 (0.98) |
| **≥80** | 14 (4.28%) | 13.8 (0.23) | 17.6 (0.27) | 23.5 (0.39) | 36.4 (0.63) | 52.1 (0.86) | 1 (0.30%) | - | - | - | - | - |
|  |  | *Isokinetic Shoulder Flexion* *at 60º/s [N·m] (Relative Isokinetic Shoulder Flexion at 60º/s [N·m/kg])* | | | | | | | | | | |
| **60-64** | 59 (18.04%) | 19.8 (0.30) | 27.2 (0.45) | 39.5 (0.61) | 44.3 (0.68) | 52.9 (0.77) | 5 (1.52%) | 49.4 (0.64) | 49.9 (0.65) | 53.2 (0.75) | 65.9 (0.85) | - |
| **65-69** | 79 (24.15%) | 16.7 (0.27) | 23.9 (0.34) | 36.1 (0.54) | 44.7 (0.65) | 50.6 (0.77) | 32 (9.78%) | 34.2 (0.47) | 42.2 (0.54) | 67.7 (0.81) | 79.6 (0.96) | 94.7 (1.10) |
| **70-74** | 61 (18.65%) | 16.2 (0.25) | 24.3 (0.34) | 32.6 (0.48) | 38.6 (0.58) | 47.7 (0.73) | 29 (8.86%) | 29.6 (0.42) | 41.0 (0.52) | 52.8 (0.68) | 64.4 (0.82) | 73.9 (0.98) |
| **75-79** | 34 (10.39%) | 18.3 (0.24) | 23.0 (0.33) | 28.8 (0.39) | 36.0 (0.50) | 52.2 (0.79) | 13 (3.97%) | 24.3 (0.35) | 35.4 (0.49) | 54.5 (0.73) | 65.7 (0.78) | 74.5 (0.90) |
| **≥80** | 14 (4.28%) | 13.5 (0.23) | 17.0 (0.26) | 23.1 (0.40) | 34.8 (0.61) | 42.9 (0.70) | 1 (0.30%) | - | - | - | - | - |
|  |  | *Isokinetic Shoulder Extension* *at 180º/s [N·m] (Relative Isokinetic Shoulder Extension at 180º/s [N·m/kg])* | | | | | | | | | | |
| **60-64** | 59 (18.04%) | 28.7 (0.40) | 33.7 (0.53) | 40.6 (0.61) | 46.6 (0.69) | 49.3 (0.75) | 5 (1.52%) | 50.0 (0.62) | 52.6 (0.67) | 56.1 (0.83) | 70.0 (0.91) | - |
| **65-69** | 79 (24.15%) | 24.7 (0.36) | 32.6 (0.48) | 39.3 (0.59) | 47.7 (0.66) | 54.5 (0.80) | 32 (9.78%) | 39.3 (0.48) | 42.6 (0.55) | 52.4 (0.64) | 62.0 (0.78) | 66.1 (0.82) |
| **70-74** | 61 (18.65%) | 26.7 (0.36) | 32.7 (0.49) | 41.4 (0.60) | 46.0 (0.71) | 51.8 (0.80) | 29 (8.86%) | 36.5 (0.45) | 39.2 (0.50) | 49.4 (0.61) | 59.8 (0.79) | 66.0 (0.97) |
| **75-79** | 34 (10.39%) | 23.1 (0.31) | 27.2 (0.37) | 35.4 (0.51) | 40.7 (0.56) | 43.5 (0.71) | 13 (3.97%) | 46.9 (0.57) | 48.9 (0.63) | 63.0 (0.78) | 65.4 (0.88) | 69.6 (0.95) |
| **≥80** | 14 (4.28%) | 16.7 (0.24) | 18.7 (0.28) | 35.9 (0.63) | 42.7 (0.65) | 52.3 (0.93) | 1 (0.30%) | - | - | - | - | - |
|  |  | *Isokinetic Shoulder Extension* *at 60º/s [N·m] Relative Isokinetic Shoulder Extension at 60º/s [N·m/kg])* | | | | | | | | | | |
| **60-64** | 59 (18.04%) | 23.4 (0.38) | 30.0 (0.44) | 33.5 (0.51) | 42.5 (0.64) | 51.2 (0.73) | 5 (1.52%) | 41.9 (0.60) | 43.3 (0.61) | 57.0 (0.71) | 72.0 (0.91) | - |
| **65-69** | 79 (24.15%) | 23.5 (0.34) | 28.4 (0.41) | 36.9 (0.54) | 48.2 (0.70) | 60.1 (0.87) | 32 (9.78%) | 35.6 (0.43) | 41.7 (0.53) | 51.5 (0.62) | 64.7 (0.78) | 77.8 (0.97) |
| **70-74** | 61 (18.65%) | 27.0 (0.36) | 29.2 (0.42) | 36.0 (0.57) | 48.3 (0.69) | 56.7 (0.82) | 29 (8.86%) | 39.5 (0.50) | 46.4 (0.58) | 52.3 (0.70) | 60.9 (0.78) | 72.4 (1.03) |
| **75-79** | 34 (10.39%) | 20.6 (0.25) | 23.4 (0.34) | 30.8 (0.39) | 38.6 (0.63) | 57.7 (0.84) | 13 (3.97%) | 40.6 (0.55) | 53.2 (0.66) | 58.7 (0.70) | 62.8 (0.90) | 71.7 (0.96) |
| **≥80** | 14 (4.28%) | 16.0 (0.23) | 21.7 (0.33) | 30.4 (0.50) | 44.3 (0.79) | 59.7 (0.89) | 1 (0.30%) | - | - | - | - | - |
| *Note for the table:* Percentiles not reported due to insufficient data are identified with “-“. Values derived from subgroups with small sample sizes (n < 10) should be interpreted with caution. N·m: Newtons per meter; kg: Kilograms. | | | | | | | | | | | | |
