# Supplementary material for: Functional fitness tests and their association with upper-limb isokinetic strength in older adults
Source: Aging Clin Exp Res. 2026 May 8;38(1):162. doi: 10.1007/s40520-026-03406-3 (PMC13357388; doi:10.1007/s40520-026-03406-3)
Supplement: Supplementary file 3 — Supplementary Material 3 [file 40520_2026_3406_MOESM3_ESM.docx]

| **Table S3.** Normative percentile values for isokinetic elbow strength (absolute and relative). | | | | | | | | | | | | |
| --- | --- | --- | --- | --- | --- | --- | --- | --- | --- | --- | --- | --- |
|  |  | *Female* | | | | |  | *Male* | | | | |
| **Percentiles** | ***n*** | **10th** | **25th** | **50th** | **75th** | **90th** | ***n*** | **10th** | **25th** | **50th** | **75th** | **90th** |
| **Age** |  | *Isokinetic Elbow Flexion* *at 180º/s [N·m] (Relative Isokinetic Elbow Flexion at 180º/s [N·m/kg])* | | | | | | | | | | |
| **60-64** | 59 (18.04%) | 10.6 (0.17) | 12.9 (0.20) | 16.4 (0.25) | 18.8 (0.28) | 22.4 (0.34) | 5 (1.52%) | 17.9 (0.27) | 22.1 (0.30) | 30.8 (0.40) | 38.2 (0.50) | - |
| **65-69** | 79 (24.15%) | 10.6 (0.16) | 13.0 (0.19) | 16.1 (0.23) | 19.2 (0.27) | 21.6 (0.34) | 32 (9.78%) | 18.9 (0.22) | 24.0 (0.29) | 26.5 (0.34) | 32.3 (0.42) | 37.3 (0.49) |
| **70-74** | 61 (18.65%) | 10.9 (0.14) | 13.3 (0.19) | 15.4 (0.22) | 17.1 (0.26) | 20.6 (0.29) | 29 (8.86%) | 17.9 (0.24) | 21.0 (0.28) | 25.8 (0.34) | 31.6 (0.40) | 39.1 (0.45) |
| **75-79** | 34 (10.39%) | 8.9  (0.13) | 10.7 (0.15) | 13.6 (0.18) | 16.5 (0.25) | 21.2 (0.30) | 13 (3.97%) | 19.7 (0.27) | 23.7 (0.28) | 25.3 (0.32) | 27.8 (0.40) | 32.3 (0.43) |
| **≥80** | 14 (4.28%) | 5.6  (0.08) | 6.7  (0.11) | 10.2 (0.17) | 15.6 (0.26) | 16.4 (0.29) | 1 (0.30%) | - | - | - | - | - |
|  |  | *Isokinetic Elbow Flexion* *at 60º/s [N·m] (Relative Isokinetic Elbow Flexion at 60º/s [N·m/kg])* | | | | | | | | | | |
| **60-64** | 59 (18.04%) | 10.3 (0.16) | 14.3 (0.20) | 17.0 (0.24) | 20.7 (0.31) | 25.5 (0.41) | 5 (1.52%) | 20.5 (0.30) | 24.2 (0.33) | 29.0 (0.38) | 36.0 (0.46) | - |
| **65-69** | 79 (24.15%) | 11.6 (0.17) | 14.5 (0.21) | 17.5 (0.24) | 20.3 (0.31) | 23.7 (0.37) | 32 (9.78%) | 20.5 (0.25) | 26.4 (0.34) | 32.1 (0.39) | 37.3 (0.47) | 47.3 (0.58) |
| **70-74** | 61 (18.65%) | 10.0 (0.15) | 13.2 (0.20) | 16.8 (0.24) | 19.4 (0.28) | 23.8 (0.35) | 29 (8.86%) | 19.4 (0.27) | 24.8 (0.32) | 30.3 (0.40) | 35.8 (0.45) | 39.2 (0.50) |
| **75-79** | 34 (10.39%) | 8.7  (0.12) | 12.2 (0.17) | 14.1 (0.20) | 20.0 (0.27) | 23.9 (0.36) | 13 (3.97%) | 19.4 (0.28) | 24.0 (0.31) | 27.8 (0.34) | 35.2 (0.46) | 39.4 (0.47) |
| **≥80** | 14 (4.28%) | 3.7  (0.07) | 10.6 (0.15) | 13.7 (0.22) | 17.6 (0.29) | 19.2 (0.34) | 1 (0.30%) | - | - | - | - | - |
|  |  | *Isokinetic Elbow Extension* *at 180º/s [N·m] (Relative Isokinetic Elbow Extension at 180º/s [N·m/kg])* | | | | | | | | | | |
| **60-64** | 59 (18.04%) | 21.1 (0.34) | 25.1 (0.39) | 33.1 (0.48) | 38.4 (0.57) | 40.8 (0.63) | 5 (1.52%) | 26.9 (0.34) | 34.9 (0.49) | 50.0 (0.62) | 53.5 (0.70) | - |
| **65-69** | 79 (24.15%) | 18.2 (0.28) | 24.0 (0.36) | 32.6 (0.47) | 37.3 (0.55) | 42.9 (0.62) | 32 (9.78%) | 34.9 (0.41) | 43.9 (0.55) | 52.6 (0.65) | 58.9 (0.75) | 63.9 (0.77) |
| **70-74** | 61 (18.65%) | 18.1 (0.26) | 24.7 (0.33) | 29.2 (0.45) | 35.5 (0.53) | 37.8 (0.58) | 29 (8.86%) | 30.0 (0.38) | 36.2 (0.47) | 44.6 (0.53) | 50.9 (0.68) | 55.0 (0.72) |
| **75-79** | 34 (10.39%) | 18.1 (0.28) | 22.0 (0.31) | 29.3 (0.38) | 33.6 (0.44) | 38.5 (0.59) | 13 (3.97%) | 40.1 (0.51) | 41.5 (0.55) | 48.6 (0.63) | 51.7 (0.68) | 62.5 (0.76) |
| **≥80** | 14 (4.28%) | 18.3 (0.32) | 22.1 (0.33) | 24.3 (0.37) | 27.8 (0.47) | 31.6 (0.54) | 1 (0.30%) | - | - | - | - | - |
|  |  | *Isokinetic Elbow Extension* *at 60º/s [N·m] (Relative Isokinetic Elbow Extension at 60º/s [N·m/kg])* | | | | | | | | | | |
| **60-64** | 59 (18.04%) | 24.4 (0.37) | 30.1 (0.48) | 37.0 (0.54) | 42.9 (0.64) | 46.7 (0.70) | 5 (1.52%) | 31.4 | 38.9 | 59.0 | 66.9 | - |
| **65-69** | 79 (24.15%) | 18.6 (0.32) | 29.3 (0.41) | 36.6 (0.53) | 42.1 (0.61) | 47.8 (0.68) | 32 (9.78%) | 42.7 | 50.9 | 59.2 | 71.3 | 76.8 |
| **70-74** | 61 (18.65%) | 23.3 (0.31) | 28.4 (0.41) | 33.6 (0.47) | 39.3 (0.61) | 44.5 (0.70) | 29 (8.86%) | 38.4 | 41.8 | 51.3 | 56.3 | 71.5 |
| **75-79** | 34 (10.39%) | 19.8 (0.30) | 24.5 (0.33) | 32.0 (0.43) | 37.8 (0.47) | 42.8 (0.69) | 13 (3.97%) | 41.4 | 51.6 | 60.6 | 65.3 | 68.0 |
| **≥80** | 14 (4.28%) | 19.9 (0.33) | 23.5 (0.37) | 26.0 (0.44) | 36.3 (0.53) | 39.0 (0.63) | 1 (0.30%) | - | - | - | - | - |
| *Note for the table:* Percentiles not reported due to insufficient data are identified with “-“. Values derived from subgroups with small sample sizes (n < 10) should be interpreted with caution. N·m: Newtons per meter; kg: Kilograms. | | | | | | | | | | | | |
