# Supplementary material for: Functional fitness tests and their association with upper-limb isokinetic strength in older adults
Source: Aging Clin Exp Res. 2026 May 8;38(1):162. doi: 10.1007/s40520-026-03406-3 (PMC13357388; doi:10.1007/s40520-026-03406-3)
Supplement: Supplementary file 4 — Supplementary Material 4 [file 40520_2026_3406_MOESM4_ESM.docx]

| **Table S4.** Associations between functional fitness tests and upper-limb isokinetic strength in women. | | | | |
| --- | --- | --- | --- | --- |
| **Isokinetic upper-limb tests** | **Correlation coefficient (r)** | | | |
|  | *Up-and-Go Test* | *30-second Chair Stand* | *30-second Arm Curl* | *6-min Walk Test* |
| Shoulder flexion 180º/s | -0.19**  (N.S.) | 0.18**  (N.S.) | 0.27**  (0.19**) | 0.37**  (0.26**) |
| Shoulder flexion 60º/s | -0.14*  (N.S.) | 0.19**  (0.13*) | 0.24**  (0.17**) | 0.34**  (0.23**) |
| Shoulder extension 180º/s | -0.20**  (N.S) | N.S.  (N.S) | 0.18**  (N.S.) | 0.17**  (N.S.) |
| Shoulder extension 60º/s | N.S.  (N.S.) | N.S.  (N.S.) | N.S.  (N.S.) | N.S.  (N.S.) |
| Elbow flexion 180º/s | N.S.  (N.S.) | N.S.  (N.S.) | 0.20**  (N.S.) | N.S.  (-0.14*) |
| Elbow flexion 60º/s | N.S.  (N.S.) | N.S.  (N.S.) | 0.14*  (N.S.) | N.S.  (N.S.) |
| Elbow extension 180º/s | -0.17**  (N.S.) | 0.19**  (0.15*) | 0.26**  (0.19**) | 0.26**  (0.16*) |
| Elbow extension 60º/s | -0.17**  (N.S.) | 0.15*  (N.S.) | 0.28**  (0.21**) | 0.29**  (0.19**) |
| Relative shoulder flexion 180º/s | -0.28**  (-0.19*) | 0.26**  (0.21**) | 0.27**  (0.20**) | 0.46**  (0.37**) |
| Relative shoulder flexion 60º/s | -0.22**  (-0.13*) | 0.26**  (0.22**) | 0.24**  (0.17**) | 0.42**  (0.33**) |
| Relative shoulder extension 180º/s | -0.28**  (-0.23**) | N.S.  (N.S.) | 0.17**  (N.S.) | 0.28**  (0.21**) |
| Relative shoulder extension 60º/s | -0.20**  (-0.19**) | N.S.  (N.S.) | N.S.  (N.S.) | 0.13*  (N.S.) |
| Relative elbow flexion 180º/s | -0.22**  (N.S.) | N.S.  (N.S.) | 0.21**  (0.13**) | 0.18**  (N.S.) |
| Relative elbow flexion 60º/s | -0.13*  (N.S.) | N.S.  (N.S.) | 0.15*  (N.S.) | 0.20**  (N.S.) |
| Relative elbow extension 180º/s | -0.29**  (-0.22**) | 0.31**  (0.27**) | 0.27**  (0.21**) | 0.38**  (0.31**) |
| Relative elbow extension 60º/s | -0.29**  (-0.22**) | 0.27**  (0.22**) | 0.29**  (0.23**) | 0.42**  (0.35**) |
| Data in parentheses show partial correlations with age as a moderator. N.S.: Non-significant. *: p<0.05; **: p<0.01. | | | | |
