# Supplementary material for: Functional fitness tests and their association with upper-limb isokinetic strength in older adults
Source: Aging Clin Exp Res. 2026 May 8;38(1):162. doi: 10.1007/s40520-026-03406-3 (PMC13357388; doi:10.1007/s40520-026-03406-3)
Supplement: Supplementary file 5 — Supplementary Material 5 [file 40520_2026_3406_MOESM5_ESM.docx]

| **Table S5.** Associations between functional fitness tests and upper-limb isokinetic strength in men. | | | | |
| --- | --- | --- | --- | --- |
| **Isokinetic upper-limb tests** | **Correlation coefficient (r)** | | | |
|  | *Up-and-Go Test* | *30-second Chair Stand* | *30-second Arm Curl* | *6-min Walk Test* |
| Shoulder flexion 180º/s | N.S.  (N.S.) | N.S.  (N.S.) | N.S.  (N.S.) | 0.26*  (0.26*) |
| Shoulder flexion 60º/s | N.S.  (N.S.) | N.S.  (N.S.) | N.S.  (N.S.) | 0.34**  (0.30**) |
| Shoulder extension 180º/s | N.S.  (N.S) | N.S.  (N.S.) | N.S.  (N.S.) | N.S.  (0.23*) |
| Shoulder extension 60º/s | N.S.  (N.S.) | N.S.  (N.S.) | N.S.  (N.S.) | 0.32**  (0.32**) |
| Elbow flexion 180º/s | N.S.  (N.S.) | N.S.  (N.S.) | 0.34**  (0.34**) | 0.34**  (0.31**) |
| Elbow flexion 60º/s | N.S.  (N.S.) | N.S.  (N.S.) | N.S.  (N.S.) | 0.22*  (N.S.) |
| Elbow extension 180º/s | N.S.  (N.S.) | N.S.  (N.S.) | N.S.  (N.S.) | 0.33**  (0.30**) |
| Elbow extension 60º/s | -0.28*  (-0.25*) | N.S.  (N.S.) | N.S.  (N.S.) | 0.45**  (0.43**) |
| Relative shoulder flexion 180º/s | N.S.  (N.S.) | N.S.  (N.S.) | N.S.  (N.S.) | 0.23*  (N.S.) |
| Relative shoulder flexion 60º/s | N.S.  (N.S.) | N.S.  (N.S.) | N.S.  (N.S.) | 0.31**  (0.27**) |
| Relative shoulder extension 180º/s | N.S.  (N.S.) | N.S.  (N.S.) | 0.27*  (-0.27*) | N.S.  (N.S.) |
| Relative shoulder extension 60º/s | N.S.  (N.S.) | N.S.  (N.S.) | N.S.  (N.S.) | N.S.  (N.S.) |
| Relative elbow flexion 180º/s | N.S.  (N.S.) | N.S.  (N.S.) | N.S.  (N.S.) | 0.26*  (N.S.) |
| Relative elbow flexion 60º/s | N.S.  (N.S.) | N.S.  (N.S.) | N.S.  (N.S.) | N.S.  (N.S.) |
| Relative elbow extension 180º/s | N.S.  (N.S.) | N.S.  (N.S.) | N.S.  (N.S.) | 0.25*  (N.S.) |
| Relative elbow extension 60º/s | -0.28*  (-0.25*) | N.S.  (N.S.) | N.S.  (N.S.) | 0.39**  (0.37**) |
| Data in parentheses show partial correlations with age as a moderator. N.S.: Non-significant. *: p<0.05; **: p<0.01. | | | | |
