# Supplementary material for: Functional fitness tests and their association with upper-limb isokinetic strength in older adults
Source: Aging Clin Exp Res. 2026 May 8;38(1):162. doi: 10.1007/s40520-026-03406-3 (PMC13357388; doi:10.1007/s40520-026-03406-3)
Supplement: Supplementary file 6 — Supplementary Material 6 [file 40520_2026_3406_MOESM6_ESM.docx]

| **Table S6.** Full regression equations for stepwise models examining the association between functional fitness tests and upper-limb isokinetic strength | |
| --- | --- |
| **Outcome** | **Full Regression Equation** |
| *Absolute Values* | |
| Shoulder flexion 180º/s | -27.18 + (22.55×Sex) + (0.07×6MWT) + (0.47×30AC) |
| Shoulder flexion 60º/s | -23.88 + (19.51×Sex) + (0.06×6MWT) + (0.43×30AC) |
| Shoulder extension 180º/s | 11.77 + (13.22×Sex) + (0.03×6MWT) |
| Shoulder extension 60º/s | 19.78 + (17.31×Sex) |
| Elbow flexion 180º/s | 17.94 + (11.47×Sex) + (-0.22×Age) + (0.38×30AC) + (-0.36×30CS) |
| Elbow flexion 60º/s | 15.03 + (13.76×Sex) + (-0.18×Age) + (0.30×30AC) + (-0.32×30CS) |
| Elbow extension 180º/s | -6.48 + (15.40×Sex) + (0.03×6MWT) + (0.33×30AC) |
| Elbow extension 60º/s | -13.70 + (19.83×Sex) + (0.04×6MWT) + (0.33×30AC) |
| *Relative Values* | |
| Shoulder flexion 180º/s | -0.30 + (0.001×6MWT) + (0.19×Sex) + (0.005×30AC) |
| Shoulder flexion 60º/s | 0.15 + (0.001×6MWT) + (0.18×Sex) + (-0.004×Age) |
| Shoulder extension 180º/s | 0.21 + (0.001×6MWT) + (0.08×Sex) |
| Shoulder extension 60º/s | 0.22 + (0.13×Sex) + (0.000×6MWT) |
| Elbow flexion 180º/s | 0.31 + (0.11×Sex) + (-0.003×Age) + (0.002×30AC) |
| Elbow flexion 60º/s | 0.33 + (0.14×Sex) + (-0.003×Age) |
| Elbow extension 180º/s | -0.54 + (0.13×Sex) + (0.001×6MWT) + (0.008×30CS) |
| Elbow extension 60º/s | -0.13 + (0.17×Sex) + (0.001×6MWT) + (0.006×30CS) |
| Sex was coded as 1 = women and 2 = men. Only variables retained in the final stepwise models are included in the equations. UGT: Up-and-Go Test; 30CS: 30-Second Chair Stand Test; 30AC: 30-Second Arm Curl Test; 6MWT: 6-Minute Walk Test | |
